# *Pf*HRP2 and *Pf*LDH antigen detection for monitoring the efficacy of artemisinin-based combination therapy (ACT) in the treatment of uncomplicated *falciparum *malaria

**DOI:** 10.1186/1475-2875-8-211

**Published:** 2009-09-07

**Authors:** Sandrine Houzé, Mainoumata Dicko Boly, Jacques Le Bras, Philippe Deloron, Jean-François Faucher

**Affiliations:** 1 Parasitology Laboratory EA209, AP-HP, Bichat-C. Bernard Hospital, Paris-Descartes University, 46 rue Henri Huchard, 75018 Paris, France; 2 Institut de Recherche pour le Développement (IRD), Mother and Child Health in the Tropics Research Unit, Cotonou, Bénin; 3 IRD, Mother and Child Health in the Tropics Research Unit, 4 avenue de l'Observatoire, 75005 Paris, France; 4 Department of Infectious Diseases, Besançon University Medical Center, 2, place Saint-Jacques, 25030 Besançon cedex, France

## Abstract

**Background:**

An assessment of the accuracy of two malaria rapid diagnostic tests (RDT) for the detection of *Plasmodium falciparum *histidine-rich protein 2 (*Pf*HRP2) or *Pf *lactate dehydrogenase (*Pf*LDH) was undertaken in children aged between six and 59 months included in an anti-malarial efficacy study in Benin.

**Methods:**

In Allada (Benin), 205 children aged 6-59 months with falciparum malaria received either artesunate-amodiaquine (ASAQ), artemether-lumefantrine (AL), or sulphadoxine-pyrimethamine (SP). Children included in the study were simultaneously followed by both RDT and high-quality microscopy for up to 42 days.

**Results:**

At the time of inclusion, *Pf*HRP2-based tests were positive in 203 children (99%) and *Pf*LDH-based tests were positive in 204 (99.5%). During follow-up, independent of the treatment received, only 17.3% (28/162) of children effectively cured were negative with the *Pf*HRP2 RDT at day 3, with a gradual increase in specificity until day 42. The specificity of antigen detection with the *Pf*LDH test was 87% (141/162) on day 3, and between 92% and 100% on days 7 to 42. A statistical difference was observed between the persistence of *Pf*HRP2 and *Pf*LDH antigenaemia during follow-up in children treated with artemisinin-based combination therapy (ACT) but not with SP.

**Conclusion:**

Although both RDTs are as sensitive as microscopy in detecting true malaria cases, the *Pf*HRP2 RDT had very low specificity during follow-up until day 28. On the other hand, the *Pf*LDH test could be used to detect failures and, therefore, to assess anti-malarial efficacy.

## Background

In response to increased anti-malarial drug resistance, artemisinin-based combination therapy (ACT) is recommended in Africa [1]. Due to the significantly higher cost of ACT than drugs formerly used, such as chloroquine and sulphadoxine-pyrimethamine (SP), strong emphasis has been placed on the importance of avoiding any unnecessary use of ACT, so as to minimize opportunities for the development of parasite drug resistance to it. Since clinical diagnosis is non-specific, laboratory confirmation is essential for accurate diagnosis of malaria. However, although microscopy is still considered the gold standard, it is unavailable in many endemic areas. An alternative is the use of a rapid diagnostic test (RDT) that is simple to perform, requires neither equipment nor electricity, and provides a result within 15 to 20 minutes [2].

The numerous commercially available RDTs fall into two categories [3]. One group of RDTs detects histidine-rich protein 2 (*Pf*HRP2), uniquely synthesized by *Plasmodium falciparum *present in the bloodstream of infected individuals. The second group of RDTs detects parasite lactate dehydrogenase (*Psp*LDH), an enzyme produced by all four *Plasmodium *species responsible for human malaria, and may also detect species-specific LDH, such as *Pf*LDH, specific of *P. falciparum*, or *Pv*LDH, specific of *Plasmodium vivax*.

Although, *Pf*HRP2 test kits have generally shown higher sensitivity for *P. falciparum *and can be less costly than the *Psp*LDH alternative, studies have shown that *Pf*HRP2 remains in the bloodstream for an extended time following successful eradication of the parasite, thus contributing to false-positive results and limiting specificity [4]. A recent study has shown day-14 and day-21 *Pf*HRP2 false-positivity rates of 98.2% and 94.6%, respectively [5]. In order to monitor the success of anti-malarial drug therapy particularly in the presence of fever, it is critical to accurately differentiate between a new episode of malaria and another cause. Due to the increase in drug-resistant infections, it has been suggested that *Psp*LDH-based tests are more useful for this purpose, since they become negative soon after parasite clearance from the blood [4,6]. However, the presence of gametocytes could give a false-positive result, especially if high gametocytaemia persists [7]. ACT could modify this situation because of its rapid effect and anti-gametocyte action [8].

Therefore, in conjunction with an anti-malarial efficacy study underway in Benin, the accuracy of two commercial malaria RDTs in diagnosing and monitoring malaria treatment was evaluated. The RDTs studied were Immunoquick^® ^Malaria (Biosynex, Strasbourg, France), a *Pf*HRP2-based test, and Optimal^® ^IT (Diamed, Cressier, Switzerland), a *Psp*LDH-*Pf*LDH-based test. The results obtained with these two tests were compared with those obtained using a thick blood film. The kinetics of the disappearance of *Pf*HRP2 and *Pf*LDH antigens following treatment were also studied. The performances of both RDTs were assessed during follow-up.

## Methods

### Study site

The RDT assessment took place concurrently with a 42-day ACT efficacy trial that conformed to WHO guidelines, which has been described elsewhere [9]. In brief, Benin is an African country in which malaria is highly endemic and seasonal, with peaks during the low (March to May) and high (September to November) rainy seasons. The study site consisted of two small towns, Allada and Sekou, in the south of Benin, 50 km north of Cotonou, the capital. *Plasmodium falciparum *accounts for 95% of the *Plasmodium *species in this region. Study approval was obtained from the ethics committee of the Benin National Malaria Program.

### Study design

The RDT assessment was carried out between April and November 2007. After screening febrile children aged between six and 59 months, those confirmed with *falciparum *malaria, who met inclusion criteria for the ACT efficacy study were recruited after their guardians had provided informed written consent. Two rapid diagnostic tests were conducted, the *Pf*HRP2-based Immunoquick^® ^Malaria (Biosynex, Strasbourg, France) and the *Psp*LDH-*Pf*LDH-based Optimal^® ^IT (Diamed, Cressier, Switzerland).

Children were randomized to receive either artesunate + amodiaquine (ASAQ), artemether + lumefantrine (AL), or sulphadoxine-pyrimethamine (SP). Most of the children were followed up to assess both anti-malarial efficacy and duration of false-positive RDT results, as defined by positive RDT and continued negative microscopy after anti-malarial treatment. For purposes of RDT assessment, follow-up was conducted by clinical examination and finger-prick blood samples on days 3, 7, 14, 21, 28, 35, and 42 post-treatment, or on any other day if the child was unwell.

### Laboratory procedures

Blood films and rapid tests were carried out on the same finger-prick blood sample. A thick blood film for microscopic detection of parasites was prepared and stained with 10% Giemsa. The thick films were read by experienced technicians. Asexual parasites were counted against 200-500 leukocytes and converted to the number of parasites per unit volume, assuming 8,000 leukocytes/μL blood [10]. Gametocytes were reported after examining 100 microscopic fields. Slides were considered negative if no parasites were detected after viewing 100 microscope fields. Microscopists were unaware of patient treatment allocation.

Internal quality control included a blind second reading of a portion of the slides as follows: all slides taken during days 0 and 3, all positive slides after day 3, and 20% of negative slides. All day-28 and day-35 negative slides were reread. In case of a discrepancy, two technicians re-examined the slides together and mutually decided on the reading. External controls were conducted by a Benin Ministry of Health government reference laboratory in the provincial capital, Cotonou.

The RDT tests were obtained directly from the manufacturers and stored in their original packaging at room temperature. Package desiccant quality was checked before using the tests [11]. All RDTs were labeled with patient ID numbers, carried out, and interpreted according to the manufacturers' instructions. All RDTs were read visually, and a control line was established for the purpose of validating the test. For cases in which the control line did not appear, the result was considered invalid and the test repeated. For the *Pf*HRP2-based test, results were recorded 15 minutes after placing the strip into six drops (300 μL) of buffer. The presence of both the control and test lines indicated a positive result for *P. falciparum*; the presence of the control line alone indicated a negative result. Results were recorded after 20 minutes for the *Pf*LDH-based test. For *Pf*LDH, the presence of the test line indicated a positive result for *P. falciparum*, and the presence of the test line for *Psp*LDH indicated a positive result for *Plasmodium *species, including *P. falciparum*. The technician recording the RDT result was unaware of the corresponding microscopy results. Internal quality control included an immediate blind second reading of 100% of the RDTs. In case of disagreement, two technicians re-examined the RDT together and decided on the reading.

### Analysis

Specificity, sensitivity, and predictive values of the RDT [with a confidence interval of 95% (95% CI)] were estimated using microscopy as the denominator for comparison. Sensitivity was defined as the percentage of positive tests among the total number of positive blood slides. Specificity was defined as the percentage of negative tests among the total number of negative blood slides. The positive predictive value (PPV) was defined as the percentage of positive blood slides among the total number of positive tests. The negative predictive value (NPV) was defined as the percentage of negative blood slides among the total number of negative tests. The percentage of false-positive (FP) tests was defined as the percentage of tests that remained positive during follow-up once the blood sample was negative by microscopy for asexual-stage parasites. The presence of gametocytes alone was not considered to be a positive microscopy result. Categorical variables were compared using the Fisher or X_2 _exact tests, in accordance with the number of patients. A *p *value of <0.05 was considered statistically significant.

## Results

### Characteristics of study subjects

Between March and September 2007, 205 children presenting with fever and a *P. falciparum *parasitaemia above 1,000/μL were included in the study. Parasite densities ranged between 1,000 and 525,000 parasites/μl.

The children were divided into three groups, according to their treatment allocation: 81 were treated with the AL combination, 80 with the ASAQ combination, and 44 with the SP combination. During the six-week follow-up period, 80 children were followed to day 42 and 40 children were followed to day 28. Among the children included in the three groups, 72 were given additional treatment within 35 days, due to malaria recrudescence or re-infection (and were excluded from the RDT study after this second treatment), and 13 children were lost to follow-up. Detailed results of the efficacy study have been published [9]. Seven gametocyte carriers were diagnosed during the follow-up (two in the AL treatment group, one in the ASAQ treatment group, and four in the SP treatment group.)

### RDT validity

On day 0, similar sensitivities were observed with both RDTs. The *Pf*HPR2-based tests were positive for 203 children (99%, [95% CI: 96.5-99.7]) and the *Pf*LDH-based tests were positive for 204 (99.5%, [95% CI: 97.3-99.9]). *Psp*LDH and *Pf*LDH detection yielded similar results during the study. Since only children with *P. falciparum *malaria were included in the study, only *Pf*LDH detection results are presented and compared with *Pf*HRP2 detection.

Relatively few samples were positive by microscopy during the days following treatment. Thirty-five samples were positive on day 3, seven on days 7 and 14, 16 on day 21, 15 on day 28, 11 on day 35, and three on day 42 (Table 1). Sensitivity decreased with day of follow-up for both the *Pf*HPR2-based test (from 100% on day 3 to 67% on day 42) and the *Pf*LDH-based test (from 80% on day 3 to 67% on day 42) (Table 1). On the other hand, specificity increased during follow-up, going from 17% on day 3 to 95% on day 42 for the *Pf*HPR2-based test and from 87% on day 3 to 100% on day 42 for the *Pf*LDH-based test (Table 2). From day 3 to day 28 of follow-up, the specificity of the *Pf*LDH-based test was statistically higher than that of the *Pf*HRP2-based test (Table 2). Negative predictive values (except one) during follow-up were greater than 95% (Table 3) for both the *Pf*HPR2- and *Pf*LDH-based tests. On the contrary, positive predictive values were not satisfactory for either RDT (less than 80%), except for *Pf*LDH-based tests carried out on day-28 samples (Table 4).

**Table 1:** Sensitivity of antigen detection with rapid diagnostic tests compared to blood slide microscopy during follow-up.

**Sensitivity**				
	***Pf*LDH**		***Pf*HRP2**	

	**%**	**95% CI**	**%**	**95%CI**

D_3_	80.0 (28/35)	64.1 - 90.0	100 (35/35)	90.1 - 100.0

D_7_	71.4 (5/7)	35.9 - 91.2	85.7 (6/7)	48.7 - 97.4

D_14_	71.4 (5/7)	35.9 - 91.2	85.7 (6/7)	48.7 - 97.4

D_21_	100 (16/16)	80.6 - 100.0	87.5 (14/16)	64.0 - 96.5

D_28_	86.7 (13/15)	62.1 - 96.3	86.7 (13/15)	62.1 - 96.3

D_35_	81.8 (9/11)	52.3 - 94.9	81.8 (9/11)	52.3 - 94.9

D_42_	66.7 (2/3)	20.8 - 93.9	66.7 (2/3)	20.8 - 93.9

**Table 2:** Specificity of antigen detection with rapid diagnostic tests compared to blood slide microscopy during follow-up.

**Specificity**				
	***Pf*LDH**		***Pf*HRP2**	

	**%**	**95%CI**	**%**	**95%CI**

D_3_	87.1 (141/162) ***	81.0 - 91.4	17.3 (28/162)	12.2 - 23.8

D_7_	92.0 (151/164) ***	86.9 - 95.3	29.9 (49/164)	23.4 - 37.3

D_14_	96.1 (150/156) ***	91.9 - 98.2	55.8 (87/156)	47.9 - 63.3

D_21_	96.5 (137/142) ***	92.1 - 98.5	73.2 (104/142)	65.4 - 79.8

D_28_	97.6 (122/125) ***	93.1 - 98.2	73.6 (92/155)	65.2 - 80.5

D_35_	97.6 (81/83) NS	91.6 - 99.3	95.2 (79/83)	88.2 - 98.1

D_42_	100.0 (77/77) NS	95.2 - 100.0	94.8 (73/77)	87.4 - 98.0

***: p < 0.0001; NS: p > 0.05

**Table 3:** Negative predictive value of antigen detection with rapid diagnostic tests compared to blood slide microscopy during follow-up.

**Negative predictive value**				
	***Pf*LDH**		***Pf*HRP2**	

	**%**	**95%CI**	**%**	**95%CI**

D_3_	95.3 (141/148)	90.6 - 97.7	100 (28/28)	87.9 - 100.0

D_7_	98.1 (151/154)	95.4 - 99.6	98.0 (49/50)	89.5 - 99.7

D_14_	98.7 (150/152)	95.3 - 99.6	98.9 (87/88)	93.8 - 99.8

D_21_	100 (137/137)	97.3 - 100.0	98.1 (104/106)	93.4 - 99.5

D_28_	98.4 (122/124)	94.3 - 99.6	97.9 (92/94)	92.6 - 99.4

D_35_	97.6 (81/83)	91.6 - 99.4	97.6 (81/83)	91.6 - 99.3

D_42_	98.7 (77/78)	93.1 - 99.8	98.7 (73/74)	92.7 - 99.8

**Table 4:** Positive predictive value of antigen detection with rapid diagnostic tests compared to blood slide microscopy during follow-up.

**Positive predictive value**				
	***Pf*LDH**		***Pf*HRP2**	

	**%**	**95% CI**	**%**	**95%CI**

D_3_	57.1 (28/49)	43.3 - 70.0	20.7 (35/169)	15.3 - 27.4

D_7_	27.8 (5/18)	12.5 - 50.9	5.0 (6/121)	2.2 - 10.4

D_14_	45.4 (5/11)	21.3 - 72.0	8.0 (6/75)	3.7 - 16.4

D_21_	76.2 (16/21)	54.9 - 89.4	26.9 (14/52)	16.8 - 40.2

D_28_	81.2 (13/16)	57.0 - 93.4	28.3 (13/46)	17.3 - 42.6

D_35_	81.8 (9/11)	52.3 - 94.9	69.2 (9/13)	42.4 - 87.3

D_42_	100.0 (2/2)	34.2 - 100.0	66.7 (4/6)	30.0 - 90.3

### Effect of parasite density and PfHRP-2 persistence

**Figure 1:**
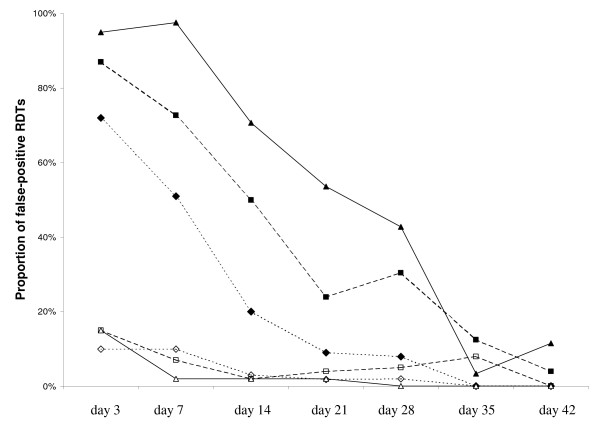
**Proportion of children with false-positive *Pf*HRP2-based test or *Pf*LDH-based test during follow-up, stratified by day-0 parasite density**. *Pf*HRP2: parasite density: black triangle: 50,000 - 525,000 p/μL; black square: 10,000 - 50,000 p/μl; black diamond: 1,000 - 10,000 p/μl. *Pf*LDH: parasite density: white triangle: 50,000 - 525,000 p/μL; white square: 10,000 - 50,000 p/μl; white diamond: 1,000 - 10,000 p/μl.

Figure 1 shows the proportion of false-positive results obtained during follow-up for *Pf*HRP2 and *Pf*LDH antigen detection as a function of parasite density at screening. Day-0 parasitaemia (number of parasites/μl) was stratified into low (1,000 - 10,000; n = 78), middle (10,000 - 50,000; n = 75), and high (50,000 - 525,000 (n = 52). The proportion of children with low parasite density was lower than the proportion with high parasite density from day 7 to day 35 for *Pf*HRP2-positive antigen detection. Twenty-eight days after effective treatment, 8% (4/50; 95% CI 3.1 - 18.9) of the children with low parasite density had *Pf*HRP2 false-positive results, compared to 42.9% (15/35; 95% CI 28.0 - 59.1) of those with high parasite density (p = 0.0004). In contrast, on any day during follow-up, the proportion of false-positive *Pf*LDH antigen detection results was low and similar in all three groups (Figure 1).

### Effect of treatment on the kinetics of antigen disappearance

**Figure 2:**
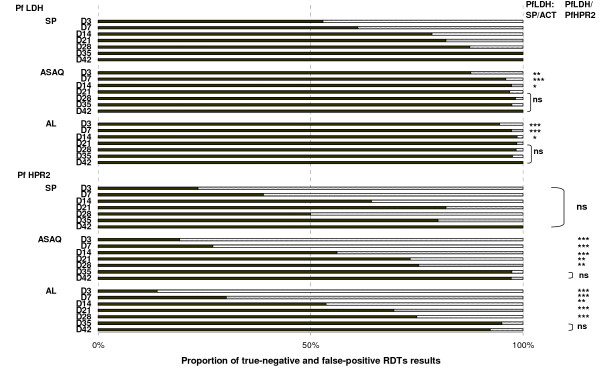
**Proportion of true-negative and false-positive results for *PfHPR2 *and *PfLDH *according to the treatment during follow-up**. (SP: sulphadoxine-pyrimethamine; ASAQ: artesunate-amodiaquine, AL: artemether-lumefantrine). Statistical analysis of antigen detection specificities for ACT compared to SP and for *Pf*LDH compared to *Pf*HPR2 are shown on the right: ***: *p*0.001; **: *p *< 0.01: *: *p *< 0.05, ns: not significant.

Independent of the treatment received, *Pf*HRP2 antigen persisted until day 35; indicating a high proportion of false-positive results with the *Pf*HPR2-based test (Figure 2). The proportion of false positive results was unrelated to the treatment given. The specificity of *Pf*LDH detection differed among treatment allocations. After SP treatment, there was no statistical difference between *Pf*HRP2 and *Pf*LDH persistence. After AL and ASAQ treatments, fewer false positive results were observed with *Pf*LDH than with *Pf*HRP2 from day 3 to day 28, (all p < 0.01). Moreover, after SP treatment, the proportion of false-positive results obtained with the *Pf*LDH-based test was high until day 14 (Figure 2). The specificity of the *Pf*LDH-based test was higher after ACT than after SP treatment from day 3 to day 14 (all p < 0.05). No correlation was found between the presence of gametocytes and the duration of RDT positivity, except on day 7, when three among the 13 results were false-positive (13/164) with the *Pf*LDH-based test in gametocyte carriers.

### Evaluation of false-negative results

Possible reasons for negative results included low parasite density and/or deficient in antigen parasites for both *Pf*HPR2 and *Pf*LDH detection. PCR-amplified exon 2 *Pf*HRP2 fragments were obtained on day 0 from the two negative isolates with the *Pf*HPR2-based test; therefore, the parasites were not antigen-deficient. False-negative results obtained during follow-up with both the *Pf*HPR2- and *Pf*LDH-based tests could be explained by low parasite densities, comprising between 16 and 800 parasites/μl.

## Discussion

The results of this study reveal that the specificity of the *Pf*LDH-based test is below 90% only for a short time, up to seven days after effective treatment. During days 3 to 28 post-treatment, *Pf*LDH detection was more specific than *Pf*HRP2, as has been demonstrated in other studies [12,13]. Since the production of both *Psp*LDH and *Pf*LDH are both related to parasite viability [6], the rapid disappearance of *Pf*LDH in blood after treatment may be due to parasite death following adequate treatment [14]. In addition, this study revealed a drug-related effect on the duration of *Pf*LDH-based test positivity after treatment. Following ACT, the persistence of *Pf*LDH was of shorter duration than that of *Pf*HRP2. In addition, *Pf*LDH false-positivity lasted longer following SP treatment than with ACT (until day 14). The rapid action of artemisinin derivatives on all parasite blood stages could be the reason for the fast clearance of *Pf*LDH in blood [15].

The duration of false positivity of the *Pf*HPR2-based test can be as long as 28 days after effective treatment. As mentioned above, past evidence strongly suggests that, to a large extent, this is due to the increased time it takes to clear *Pf*HPR2 from the blood following *P. falciparum *clearance. The duration of false positivity observed in this study with the *Pf*HPR2-based test has been correlated to higher parasite density on admission. Since secretion of the protein is proportional to parasite numbers [16], a higher parasite density on admission would require an extended period of time for *Pf*HPR2 to be cleared from blood. The results of the present study further support this point, indicating a strong correlation between the duration of *Pf*HPR2-based test positivity and parasite density at admission. Although the mechanism of *Pf*HRP2 clearance is not well understood, there are several explanations for its long persistence following adequate therapy. As previously suggested by Singh and Shukla [17], the present study was designed to investigate whether the persistence of *Pf*HRP2 in blood after efficacious treatment was influenced by the treatment. Indeed, artemisinin derivatives are active during the *Plasmodium *ring stage, leading to the rapid disappearance of parasites in blood [15]. The production of *Pf*HRP2, which is generated at all *Plasmodium *stages, could be stopped earlier with ACT than with SP, and a parallel quick clearance of *Pf*HRP2 could occur [18]. However, the results of the study did not support this hypothesis, since no difference was observed in the persistence of *Pf*HRP2 after treatment with either artesunate or artemether (fast-acting artemisinin compounds) or SP. In addition, *Pf*HRP2 false-positives determined by comparison with microscopy results are observed in identical proportions in all three treatment groups during follow-up.

The reference method in this study was the microscopy because according WHO recommendations, efficacy is defined according to clinical and microscopy criteria [10]. Probably, PCR on account of its high sensitivity may have given slightly different results but the efficiency of PCR to manage treatment follow-up was not yet demonstrated.

*Pf*HRP2 is also released by early gametocytes [19]. Therefore, the persistence of immature gametocytes in blood after successful therapy could result in the persistence of *Pf*HPR2 positivity [20]. In the studied population, whatever the treatment group, only a few gametocyte carriers were detected. This study did not allow a conclusion regarding the existence of an association between gametocytaemia and duration of *Pf*HRP2 or *Pf*LDH detection positivity, due to the small number gametocytes carriers. The ACT effect on gametocytes did not account for the shorter persistence of *Pf*HRP2 compared to the effect of SP therapy.

The results of this study reveal high *Pf*HPR2- and *Pf*LDH-based test sensitivities compared with microscopy at the time of screening. In contrast with other studies [21,22], a high sensitivity was observed with the *Pf*LDH-based test, equivalent to that of the *Pf*HPR2-based test. Previous studies may have been carried out using reagents whose quality had been altered by heat and/or humidity. Reagent quality has now been improved and conservation is better, even under drastic conditions [23]. The high parasite levels in the children included in the study could also explain this result [24]. Discrepancies were found in three samples: two were positive with the *Pf*HPR2-based test, but negative with *Pf*LDH-based test, and the third was negative with the *Pf*HPR2-based test but positive with *Pf*LDH-based test. No alteration was found by PCR in the DNA of *Pf*HRP2 and *Pf*LDH genes that could explain the false-negative RDT results obtained for these samples [25,26].

In this study, for each day of follow-up, the sensitivities of the tests to detect failures were between 67% and 100%, but the numbers of relapsing patients were low (between 3 and 16), and the parasitaemia in positive samples not detected by the tests was low. Whereas, for the diagnosis, the performance of both RDT tests used in the study is good as demonstrated by WHO with a sensitivity of 100% for the *Pf*HPR2-based test and 96.2% for the *Pf*LDH-based test against *P. falciparum *samples at parasites densities more than 2,000/μl [24]. However, given its high specificity, the predictive positive values of the *Pf*LDH-based test during follow-up were sufficient to consider a positive result to be a strong presumption of relapse, especially after ACT treatment.

## Conclusion

In conclusion, Optimal^® ^IT allows detection of both *Psp*LDH and *Pf*LDH and can be used during follow-up of an *in vivo *drug sensitivity test to confirm the effectiveness of anti-malarial treatment, especially after ACT or other quick-acting drugs, or to confirm suspected relapse. Immunoquick^® ^Malaria allows detection of *Pf*HRP2 and is a good test for ruling out a diagnosis of malaria, but should not be used during follow-up prior to day 35.

## Competing interests

The authors declare that they have no competing interests.

## Authors' contributions

SH, JLB, PD, and JFF conceived and designed the study. SH, MDB, and JFF participated in data collection. SH and JLB participated in the data analysis. All authors participated in the writing of the manuscript, read, and approved the final manuscript.
